# A Global Calibration Method for Widely Distributed Cameras Based on Vanishing Features

**DOI:** 10.3390/s16060838

**Published:** 2016-06-08

**Authors:** Xiaolong Wu, Sentang Wu, Zhihui Xing, Xiang Jia

**Affiliations:** School of Automation Science and Electrical Engineering, Beihang University, Beijing 100191, China; woost@sina.com (S.W.); zhxing@buaa.edu.cn (Z.X.); lhfy607@163.com (X.J.)

**Keywords:** widely-distributed vision sensors, global calibration, parallel line, vanishing points, vanishing lines, pose estimation

## Abstract

This paper presents a global calibration method for widely distributed vision sensors in ring-topologies. Planar target with two mutually orthogonal groups of parallel lines is needed for each camera. Firstly, the relative pose of each camera and its corresponding target is found from the vanishing points and lines. Next, an auxiliary camera is used to find the relative poses between neighboring pairs of calibration targets. Then the relative pose from each target to the reference target is initialized by the chain of transformations, followed by nonlinear optimization based on the constraint of ring-topologies. Lastly, the relative poses between the cameras are found from the relative poses of calibration targets. Synthetic data, simulation images and real experiments all demonstrate that the proposed method is reliable and accurate. The accumulated error due to multiple coordinate transformations can be adjusted effectively by the proposed method. In real experiment, eight targets are located in an area about 1200 mm × 1200 mm. The accuracy of the proposed method is about 0.465 mm when the times of coordinate transformations reach a maximum. The proposed method is simple and can be applied to different camera configurations.

## 1. Introduction

Vision sensors have advantages of flexibility and high-precision. Distributed vision sensors (DVS) are always used due to their wider fields of view (FOVs). Calibration is an important step for most camera applications. Calibration of a DVS typically has two stages: the intrinsic calibration which can be done separately and the global calibration which calculates the relative poses of the camera frames and the global coordinate frame (GCF). Then the information extracted from each camera can be integrated into the GCF. Generally, the coordinate frame of the reference camera is selected as GCF. However, vision sensors are usually widely distributed to have a better coverage. As two adjacent cameras usually have small or no overlapping FOV, the global calibration of DVS becomes of prime importance.

DVS can be calibrated by high-precision 3D measurement equipment. Lu *et al.* [1] constructed a measurement system and achieved calibration of non-overlapping DVS using two theodolites and a planar target. Calibration methods without expensive equipment have also been investigated. Peng *et al.* [2] proposed an approach omitting translation vectors between cameras, due to the loss of depth information during the camera projection [3]. It assumes that all the cameras have approximately the same optical center. However, this assumption is not appropriate when the relative distances between cameras are not small in comparison with the distances to the captured scene. In addition, feature detection and matching, such as scale invariant feature transform (SIFT) [4] is not reliable in insufficient textural environments due to lack of enough distinctive feature points [2].

Global calibration methods for DVS with overlapping FOV cannot be applied in the case of non-overlapping FOV. Most of the global calibration methods for DVS with non-overlapping FOV are based on mirror reflections [5,6,7], rigidity constraints of calibration targets [8,9,10], movements of the platform [11] and the auxiliary camera [12]. For general distributed vision sensors, it is hard to make sure that each camera has a clear sight of the targets through mirror reflections, especially in complex environments. Liu *et al.* [9] proposed a global calibration method by placing multiple targets in front of the vision sensors at least four times. Bosch *et al.* [10] use a poster to determine the relative poses of multiple cameras in two steps. It requires that different parts of the poster are observed by at least two cameras at the same time, so that SIFT features can be utilized. However, it is not flexible to use a long one-dimensional target [8], rigidly-connected targets [9] or a large area poster [10] for the calibration of widely distributed cameras. Pagel [11] achieved extrinsic calibration of a multi-camera rig with non-overlapping FOV by moving the platform. However, it required that at least two adjacent targets be visible and two cameras can see the target at the same time. Sun *et al.* [12] used an auxiliary camera to observe all the sphere targets. However, all the targets can hardly been observed by one camera at the same time due to widely distribution of the vision sensors.

Structure from motion [13] solves similar problems as that in the global calibration of DVS. The difference is that the global calibration transforms local coordinate frames into GCF, while structure from motion estimates the locations of 3D points from multiple images [14]. Fitzagibbon *et al.* [15] recover the 3D scene structure together with 3D camera positions from a closed image sequence. Compared with open sequences, the closed image sequence contains additional constraints. Zhang *et al.* [16] propose an incremental motion estimation algorithm to deal with long image sequences.

Generally, one calibration target is selected as the reference target. Compared with employing the auxiliary camera to capture all the targets in one image, capturing neighboring pairs of targets is more suitable for widely distributed cameras. The relative poses between neighboring pairs of the targets can be solved separately. Then the relative poses between each target and the reference target can be achieved by chainwise coordinate transformations. However, the error accumulates with increasing times of transformations. When dealing with DVS that provides a vision of the surrounding scene just as [2,11], vision sensors are always configured in ring-topologies to have a better coverage of the surroundings. The first sensor adjoins the end to form a closed chain. Thus a closed image sequence of neighboring targets can be acquired by the auxiliary camera.

Line features are more stable than point features in detection and matching [17]. The principle of perspective projection indicates that an infinite scene line is mapped onto an image plane as a line terminating in a vanishing point. Vanishing points and vanishing lines are the distinguish features of perspective projection [18]. Xu *et al.* [19] proposed a pose estimation method based on vanishing lines of a T-shaped target. Wang [20] used a target with three equally spaced parallel lines to estimate the rotation matrix by moving the target into at least three different positions. Wei *et al.* [21,22] calibrated a line-structured vision sensor and a binocular vision sensor by a planar target with several parallel lines. Two mutually orthogonal groups of parallel lines are common in urban environments, such as crossroads, facades of architectures and so on. They can be used as the calibration targets. Even if they are absent in the scene, targets with two mutually orthogonal groups of parallel lines can be employed.

In this paper, we focus on the calibration of widely distributed cameras in ring-topologies. A planar target with two mutually orthogonal groups of parallel lines is allocated to each camera. The vanishing line of the target plane is obtained from two vanishing points. Then the relative pose between each camera and its corresponding target is initialized and refined based on vanishing features and the known line length. A closed image sequence of neighboring pairs of calibration targets is acquired by repeated operations of the auxiliary camera. Then the relative poses between two adjacent targets can be obtained and the transformation matrix from each target to the reference target is initialized in a chainwise manner. In order to adjust the accumulated error due to the chain of transformations, a global calibration method is proposed to optimize relative poses of the targets based on the constraint of the ring-type structure. Finally, using the targets as media, the optimal relative poses between each camera and the reference camera are obtained.

The rest of the paper is organized as follows: preliminary work is introduced in Section 2. The proposed global calibration method is described in Section 3. Accuracy analysis of different factors’ effects is given in Section 4. Synthetic data, simulation images and real data experiments are carried out in Section 5. The conclusions are given in Section 6.

## 2. Preliminaries

### 2.1. Coordinate Frame Definition

In this paper, the camera coordinate frame is used as the vision sensor coordinate frame. Assuming DVS consists of *M* cameras, C*_k_*CF (1 ≤ *k* ≤ *M*) denotes the coordinate frame of camera *k*. A*_i_*CF (1 ≤ *i* ≤ *M*) denotes the coordinate frame of the auxiliary camera that captures two adjacent targets (*i*, *j*). The origins of CCF and ACF are fixed at the optical centers, respectively. I*_k_*CF (1 ≤ *k* ≤ *M*) denotes the image coordinate frame of camera *k* in pixels. The origin of ICF is fixed at the center of the image plane.

**Figure 1:**
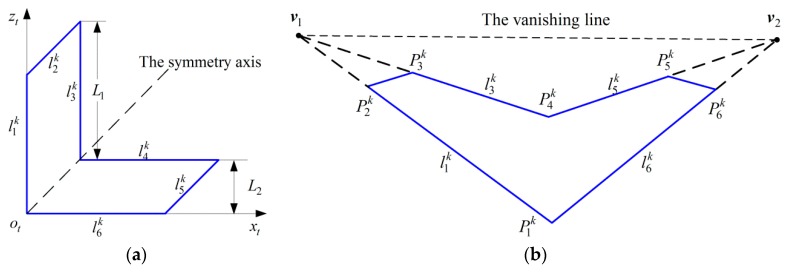
Planar target with two mutually orthogonal groups of parallel lines: (**a**) Planform of target *k*; (**b**) Perspective projection of target *k* onto the image plane.

As shown in Figure 1, the target is constructed of two mutually orthogonal groups of parallel lines with a known length *L*_1_ and the distance *L*_2_. $P_{m}^{k}$ and $l_{m}^{k}$ denote the *m*th corner point and the *m*th feature line of target *k*, 1 ≤ *m* ≤ 6. T*_k_*CF (1 ≤ *k* ≤ *M*) denotes the coordinate frame of target *k*. $l_{6}^{k}$ and $l_{1}^{k}$ coincide with the x-axis and the *z*-axis of the target, respectively. The *y*-axis is decided by the right hand rule. ECF represents the ground coordinate frame. The origin of ECF is fixed on the ground. Plane *x_e_o_e_z_e_* lies in the ground plane. The *y*-axis of ECF is decided by the right hand rule.

### 2.2. Measurement Model

In this paper, a two-dimensional image point is denoted by $p={\left[u,v\right]}^{T}$, a three-dimensional spatial point by $P={\left[X,Y,Z\right]}^{T}$. $\tilde{p}$ and $\tilde{P}$ are the corresponding homogeneous points, $\tilde{p}={\left[p^{T},1\right]}^{T}$, $\tilde{P}={\left[P^{T},1\right]}^{T}.$ The projection of a spatial point in TCF onto the image plane is described as:

(1) $$s\tilde{p}=\left[Κ|0_{3\times 1}\right]T\tilde{P},\;K=\left[\begin{array}{ccc} f_{x} & 0 & u_{0}\\ 0 & f_{y} & v_{0}\\ 0 & 0 & 1 \end{array}\right],\;T=\left[\begin{array}{cc} R_{3\times 3} & t_{3\times 1}\\ 0_{1\times 3} & 1 \end{array}\right],\;R=\left[\begin{array}{ccc} R_{11} & R_{12} & R_{13}\\ R_{21} & R_{22} & R_{23}\\ R_{31} & R_{32} & R_{33} \end{array}\right]$$

where ***K*** is the intrinsic parameter matrix, *f_x_* and *f_y_* are the equivalent focal length in horizontal and vertical directions, respectively. (*u*_0_, *v*_0_) is the principal point. ***T*** denotes the transformation matrix between targets and cameras. ***R*** is a 3 × 3 rotation matrix, ***t*** is a 3 × 1 translation vector. The rotation matrix can be expressed in terms of Y-X-Z Euler angles: yaw angle φ, pitch angle θ and roll angle ϕ:

(2) $$R\left(\varphi ,\theta ,\phi \right)=\left[\begin{array}{ccc} cos\phi cos\varphi \;+\;sin\theta sin\phi sin\varphi & cos\theta sin\phi & cos\varphi sin\theta sin\phi \;-\;cos\phi sin\varphi \\ cos\phi sin\theta sin\varphi \;-\;cos\varphi sin\phi & cos\theta cos\phi & sin\phi sin\varphi \;+\;cos\phi cos\varphi sin\theta \\ cos\theta sin\varphi & \;-sin\theta & \;cos\theta cos\varphi \end{array}\right]$$

In this paper, definitions of the transformation matrices are shown in Table 1.

## 3. The Principle of Global Calibration 

**Figure 2:**
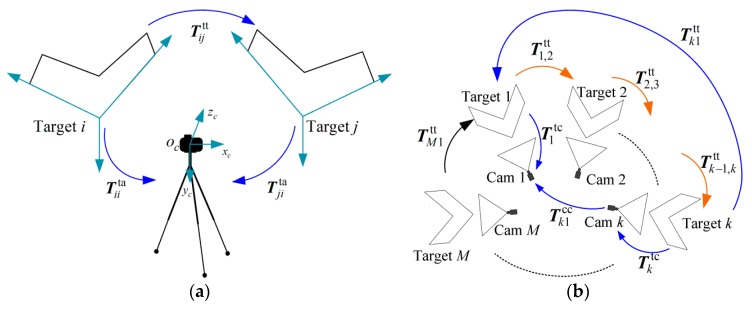
(**a**) Two adjacent targets (*i*, *j*) captured by the auxiliary camera; (**b**) The principle of the global calibration.

The principle of global calibration is shown in Figure 2. In this paper, we choose camera 1 as the reference camera as well as target 1 as the reference target. The main process of the proposed global calibration method works as follows:

1. Intrinsic calibration is done separately for each camera using the J. Bouguet Camera Calibration Toolbox based on Zhang’s calibration method [23,24].The intrinsic parameters are treated as fixed and the cameras’ poses are unchangeable during the calibration.
2. Place the planar targets in each camera’s FOV. The symmetry axis of each target is set to approximately orient to its corresponding camera. Image *I_k_* denotes target *k* captured by camera *k*. An image sequence ***I*** = {*I_k_*|1 ≤ *k* ≤ *M*} is obtained.
3. Use the auxiliary camera to capture neighboring pairs of the targets. As shown in Figure 2a, image ${\tilde{I}}_{i}$ denotes two adjacent targets (*i*, *j*) captured by the auxiliary camera. A closed image sequence $\tilde{I}$ is acquired, $\tilde{I}=\left\{{\tilde{I}}_{i}|1\leq i\leq M\right\};$ 1 ≤ *i* ≤ *M*; *j* = *i* +1 if *i* < *M*, *j* = 1 if *i* = *M*.
4. All the images are rectified to compensate for cameras’ distortion based on the intrinsic calibration results. The linear equation of parallel lines in the image plane can be obtained from the feature points extracted by Steger’s method [25].
5. Compute the transformation matrix $T_{k}^{\mathrm{tc}}$ based on the undistorted image sequence ***I***.
6. Compute the transformation matrix $T_{ij}^{\mathrm{tt}}$ of two adjacent targets based on the undistorted image sequence $\tilde{I}.$
7. Calculate the initial value of $T_{k1}^{\mathrm{tt}}$ (2 ≤ *k* ≤ *M*) by multiple coordinate transformations, as shown in Figure 2b. Then $T_{k1}^{\mathrm{tt}}$ (2 ≤ *k* ≤ *M*) is refined by the global nonlinear optimization.
8. Compute the transformation matrix $T_{k1}^{\mathrm{cc}}$ (2 ≤ *k* ≤ *M*).The calibration is completed.

### 3.1. Sloving $T_{k}^{\mathrm{tc}}$

#### 3.1.1. Feature Extraction

The feature points on a line can be extracted by Steger’s method [25], denoted by ***p****_i_* = [*u_i_*, *v_i_*]*^T^*, where 1 ≤ *i* ≤ *s*. *s* is the number of feature points. The projection of a line onto the image plane is also a straight line. The equation of a line on the image can be expressed as *au* + *bv* + *c* = 0.

Let $A={\left[\begin{array}{cccc} p_{1} & p_{2} & \ldots & p_{s} \end{array}\right]}^{T},w={\left[\begin{array}{cccc} -1 & -1 & \ldots & -1 \end{array}\right]}^{T}$, A is a s×2 matrix, w is a s×1 vector. Thus the relation of a, b and c can be obtained by the least squares method:

(3) $$\left[\begin{array}{c} a\\ b \end{array}\right]=c{\left[A^{T}A\right]}^{-1}\left[A^{T}w\right]$$

Thus, the linear equation of line $l_{m}^{k}$ can be found by the above method, where 1 ≤ *m* ≤ 6. Then the coordinates of ${\tilde{p}}_{m}^{k}$ are obtained from line intersections. As shown in Figure 1b, two vanishing points ***v***_1_ and ***v***_2_ can be found by lines $l_{1}^{k}$, $l_{3}^{k}$ and lines $l_{5}^{k}$, $l_{6}^{k}$, respectively. Then the linear equation of the vanishing line is obtained, denoted by:

(4) $$\tilde{a}u+\tilde{b}v+\tilde{c}=0$$

#### 3.1.2. Computing the Vanishing Line

As shown in Figure 1b, two groups of parallel lines converge at vanishing points ***v***_1_ and ***v***_2_ in the image plane, respectively. The line crossing ***v***_1_ and ***v***_2_ is the vanishing line. The equations of two non-parallel lines in T*_k_*CF are:

(5) $$a_{i}x+c_{i}z+d_{i}=0,\;y=0,\left(i=1,2\right)$$

where $a_{1}c_{2}-a_{2}c_{1}\neq 0.$

Let ${\tilde{V}}_{1}\text{ and }{\tilde{V}}_{2}$ be the infinite points in the two lines, ${\tilde{V}}_{i}={\left[-c_{i},0,a_{i},0\right]}^{T},\;i=1,2$ (the elaboration is given in Appendix A). We have:

(6) $$s_{i}{\tilde{v}}_{i}=\left[Κ|0_{3\times 1}\right]T_{k}^{\text{tc}}{\tilde{V}}_{i}\;\left(i=1,2\right)$$

where ***K*** denotes the intrinsic parameter matrix of camera *k*.

Combining Equations (1) and (6), we have:

(7) $${\tilde{v}}_{i}={\left[f_{x}\frac{-c_{i}R_{11}+a_{i}R_{13}}{-c_{i}R_{31}+a_{i}R_{33}}+u_{0},f_{y}\frac{-c_{i}R_{21}+a_{i}R_{23}}{-c_{i}R_{31}+a_{i}R_{33}}+v_{0},1\right]}^{T}$$

Vanishing line ***l*** can be computed by $l={\tilde{v}}_{1}\times {\tilde{v}}_{2}$. With Equation (7), we have:

(8) $$l=\left[\begin{array}{l} f_{y}(R_{23}R_{31}-R_{21}R_{33})\\ f_{x}(R_{11}R_{33}-R_{13}R_{31})\\ f_{x}f_{y}\left(R_{21}R_{13}-R_{11}R_{23}\right)+f_{x}v_{0}\left(R_{13}R_{31}-R_{11}R_{33}\right)+f_{y}u_{0}\left(R_{21}R_{33}-R_{23}R_{31}\right) \end{array}\right]$$

Combining Equations (2) and (8), the linear equation of the vanishing line is expressed as:

(9) $$\frac{\sin\phi }{f_{x}}\left(u-u_{0}\right)+\frac{\cos\phi }{f_{y}}\left(v-v_{0}\right)-\tan\theta =0$$

#### 3.1.3. Computing the Rotation Matrix of $T_{k}^{\mathrm{tc}}$

Combining Equations (4) and (9), the roll angle ϕ and pitch angle θ can be obtained:

(10) $$\left\{ \begin{array}{l} \phi =\tan^{-1}\left[f_{x}\tilde{a}/\left(f_{y}\tilde{b}\right)\right]\\ \theta =\tan^{-1}\left(-\frac{\tilde{c}}{\tilde{a}}\frac{\sin\phi }{f_{x}}-\frac{\sin\phi }{f_{x}}u_{0}-\frac{\cos\phi }{f_{y}}v_{0}\right) \end{array}\right.$$

Vanishing points are determined by the directions of the parallel lines [18], we have:

(11) $${\tilde{v}}_{i}=Kd_{i}\left(i=1,2\right)$$

where ***d****_i_* is the 3 × 1 direction vector of the line in CCF.

$l_{1}^{k}$ and $l_{6}^{k}$ coincide with the *z*-axis and the *x*-axis of T*_k_*CF, respectively. Thus:

(12) $$\left\{ \begin{array}{l} d_{1}/\left\|d_{1}\right\|=R\cdot {\left[\begin{array}{ccc} 0 & 0 & 1 \end{array}\right]}^{T}\\ d_{2}/\left\|d_{2}\right\|=R\cdot {\left[\begin{array}{ccc} 1 & 0 & 0 \end{array}\right]}^{T} \end{array}\right.$$

According to the orthogonal constraint of a Rotation matrix, the rotation matrix ***R*** of $T_{k}^{\mathrm{tc}}$ can be obtained:

(13) $$R=\left[\begin{array}{ccc} \frac{d_{2}}{\left\|d_{2}\right\|} & \frac{d_{1}\times d_{2}}{\left\|d_{1}\right\|\cdot \left\|d_{2}\right\|} & \frac{d_{1}}{\left\|d_{1}\right\|} \end{array}\right]$$

#### 3.1.4. Computing Translation Vector of $T_{k}^{\mathrm{tc}}$

$P_{7}^{k}$ is a virtual point in the target plane. As vector $\vec{P_{1}^{k}P_{2}^{k}}⊥d_{2},$ the projections of $\vec{P_{1}^{k}P_{7}^{k}}$ and $\vec{P_{2}^{k}P_{7}^{k}}$ onto the vector $d_{2}$ are equal. We have:

(14) $$d_{2}^{T}\vec{P_{1}^{k}P_{7}^{k}}=d_{2}^{T}\vec{P_{2}^{k}P_{7}^{k}}$$

Combining Equations (1) and (14), we have:

(15) $$\frac{z_{1}}{z_{2}}=\frac{d_{2}^{T}K^{-1}{\tilde{p}}_{2}^{k}}{d_{2}^{T}K^{-1}{\tilde{p}}_{1}^{k}}$$

where $z_{1}$ and $z_{2}$ are the z coordinates of $P_{1}^{k}$ and $P_{2}^{k}$ in $C_{k}\text{CF}$, respectively. ${\tilde{p}}_{1}^{k}$ and ${\tilde{p}}_{2}^{k}$ are known coordinates of the corner points.

Besides, the length of $P_{1}^{1}P_{2}^{k}$ is known:

(16) $$\left\|z_{1}K^{-1}{\tilde{p}}_{1}^{k}-z_{2}K^{-1}{\tilde{p}}_{2}^{k}\right\|=L_{1}$$

Combining Equations (15) and (16), $z_{1}$ and $z_{2}$ can be found. Thus, the coordinate of $P_{1}^{k}$ in $C_{k}\text{CF}$ is obtained. In addition, $P_{1}^{k}$ is the origin of $T_{k}\text{CF}$, the translation vector t of $T_{k}^{\text{tc}}$ can be obtained:

(17) $$t=z_{1}K^{-1}{\tilde{p}}_{1}^{k}$$

#### 3.1.5. Nonlinear Optimization

Let ${\tilde{P}}_{m}^{k}\left(1\leq m\leq 6\right)$ be the homogeneous coordinate of $P_{m}^{k}$ in $T_{k}\text{CF}$. Let ${\tilde{p}}_{m}^{k}$ be the corresponding coordinate in the image $I_{k}$. We have:

(18) $$s_{m}^{k}{\tilde{p}}_{m}^{k}=\left[Κ|0_{3\times 1}\right]T_{k}^{\text{tc}}{\tilde{P}}_{m}^{k}$$

Assuming that image points are corrupted by independently and identically distributed Gaussian noise, the maximum likelihood estimation is obtained by minimizing the sum of squared distances between the observed feature lines and the re-projected corner points. $T_{k}^{\mathrm{tc}}$ (1 ≤ *k* ≤ *M*) are refined separately by minimizing the following function using Levenberg-Marquardt algorithm [26]:

(19) $$f\left(\Omega \right)=\sum_{m=1}^{6}\left[d^{2}\left({\tilde{p}}_{m}^{k},l_{m}^{k}\right)+d^{2}\left({\tilde{p}}_{m}^{k},l_{n}^{k}\right)\right]$$

(20) $$n=\left\{ \begin{array}{l} m-1,\text{ if }m\geq 2\\ 6,\text{ if }m=1 \end{array}\right.$$

where Ω = $T_{k}^{\mathrm{tc}}$. $l_{m}^{k}$ and $l_{n}^{k}$ denotes the projections of $l_{m}^{k}$ and $l_{n}^{k}$ onto the image *I_k_*, respectively. *d*(·) denotes distances between points and lines. ***R*** of$T_{k}^{\mathrm{tc}}$ is parameterized using the Rodrigues’ formula [27].

### 3.2. Initializing $T_{k1}^{\mathrm{tt}}$

Generally, target pair (*i*, *j*) is visible in the image ${\tilde{I}}_{i}$, that:

(21) $$j=\left\{ \begin{array}{l} i+1,\text{ if }i\leq M-1\\ 1,\text{ if }i\text{ = }M \end{array}\right.$$

As shown in Figure 2a, $T_{ii}^{\mathrm{ta}}$ and $T_{ji}^{\mathrm{ta}}$ are the transformation matrices from target *i* and target *j* to the auxiliary camera, respectively. $T_{ii}^{\mathrm{ta}}$ and $T_{ji}^{\mathrm{ta}}$ can be initialized and refined separately by the methods described in Section 3.1. Then the initial value of $T_{ij}^{\mathrm{tt}}$ can be calculated by:

(22) $$T_{ij}^{\text{tt}}={\left(T_{ji}^{\text{ta}}\right)}^{-1}T_{ii}^{\text{ta}}$$

The initial value of $T_{k1}^{\mathrm{tt}}$ (2 ≤ *k* ≤ *M*) can be obtained by the minimum times of chainwise coordinate transformations:

(23) $$T_{k1}^{\text{tt}}=\left\{ \begin{array}{l} {\left[T_{k-1,k}^{\text{tt}}T_{k-2,k-1}^{\text{tt}}\cdots T_{2,3}^{\text{tt}}T_{1,2}^{\text{tt}}\right]}^{-1},\text{ if }k\leq \left(M/2\right)\\ T_{M,1}^{\text{tt}}T_{M-1,M}^{\text{tt}}\cdots T_{k+1,k+2}^{\text{tt}}T_{k,k+1}^{\text{tt}},\text{ if }k>\left(M/2\right) \end{array}\right.$$

### 3.3. Global Calibration of the Targets

According to the camera model, we have:

(24) $$\left\{ \begin{array}{l} s_{m}^{ii}{\tilde{p}}_{m}^{ii}=\left[Κ|0_{3\times 1}\right]T_{ji}^{\text{ta}}{\left(T_{j1}^{\text{tt}}\right)}^{-1}T_{i1}^{\text{tt}}{\tilde{P}}_{m}^{i}\\ s_{m}^{ji}{\tilde{p}}_{m}^{ji}=\left[Κ|0_{3\times 1}\right]T_{ii}^{\text{ta}}{\left(T_{i1}^{\text{tt}}\right)}^{-1}T_{j1}^{\text{tt}}{\tilde{P}}_{m}^{j} \end{array}\right.\;$$

where Kdenotes the intrinsic matrix of the auxiliary camera; ${\tilde{p}}_{m}^{ii}$ and ${\tilde{p}}_{m}^{ji}$ denote the reprojections of $P_{m}^{i}$ and $P_{m}^{j}$ onto the image ${\tilde{I}}_{i}$, respectively.

Assuming image points are corrupted by independent and identical Gaussian noise, $T_{k1}^{\mathrm{tt}}$ (2 ≤ *k* ≤ *M*) can be optimized by minimizing the following function using Levenberg-Marquardt algorithm [26]:

(25) $$f\left(\Omega \right)=\sum_{i=1}^{M}\sum_{m=1}^{6}\left[d^{2}\left({\tilde{p}}_{m}^{ii},l_{m}^{ii}\right)+d^{2}\left({\tilde{p}}_{m}^{ii},l_{n}^{ii}\right)+d^{2}\left({\tilde{p}}_{m}^{ji},l_{m}^{ji}\right)+d^{2}\left({\tilde{p}}_{m}^{ji},l_{n}^{ji}\right)\right]$$

where $\Omega =\left(T_{2,1}^{tt},\ldots ,\;T_{M1}^{tt}\right),\;T_{1,1}^{tt}=I_{4\times 4}$ $l_{m}^{ii}$ and $l_{m}^{ji}$ denote the projections of line $l_{m}^{i}$ and $l_{m}^{j}$ onto the image ${\tilde{I}}_{i}$, respectively. $R\text{ of }T_{k1}^{\text{tt}}\left(2\leq k\leq M\right)$ are parameterized by the Rodrigues formula. A good starting point of the optimization is provided by Equations (22) and (23). (m,n) and (i,j) subject to Equations (20) and (21), respectively.

### 3.4. Solving $T_{k1}^{\text{cc}}$

After the global calibration of the targets, the transformation matrix from each camera to the reference camera can be found:

(26) $$T_{k1}^{\text{cc}}=T_{1}^{\text{tc}}T_{k1}^{\text{tt}}{\left(T_{k}^{\text{tc}}\right)}^{-1}\;\left(2\leq k\leq M\right)$$

where $T_{k}^{\mathrm{tc}}$ (1 ≤ *k* ≤ *M*) are the results of Equation (19), $T_{k1}^{\mathrm{tt}}$ (2 ≤ *k* ≤ *M*) are the optimization results of Equation (25).

## 4. Accuracy Analysis of Different Factors’ Effects

In this section, analysis of several factors’ effects on the accuracy of the proposed method is performed by synthetic data experiments. The auxiliary camera’s intrinsic parameters are *f_x_* = *f_y_* = 512, *u*_0_ = 512, *v*_0_ = 384. The image resolution is 1024 pixel × 768 pixel.

The cameras’ positions are represented by the coordinates of the cameras’ origins in ECF. The cameras’ orientations are denoted by the Euler angles (φ, θ, ϕ) from ECF to CCF. Targets are placed on the ground for convenience. The targets’ positions are represented by the coordinates of the targets’ origins in ECF. The targets’ orientations are denoted by the yaw angle φ from positive z-axis of ECF to the symmetry axis of the target. 

*dR* and *dt* denote the 2-norm of rotation vector and translation vector differences between the calculation results and the real data. The RMS errors of *dR*, *dt* are used to evaluate the accuracy. The number of points that emulate feature lines is equal to the line length in pixels. Gaussian noise with zero mean and different noise levels is added to the image coordinates of the points of feature lines. Analysis of the factors’ effects is shown as follows.

### 4.1. Accuracy vs. the Pitch Angle of Camera Relative to the Target

The image sequence $\tilde{I}$ is acquired by the auxiliary camera. The pitch angle of the auxiliary camera relative to the target plane is one of the factors affecting the calibration accuracy. In this experiment, two adjacent targets are captured by the auxiliary camera at different pitch angles. The targets’ positions in ECF are [−450, 0, 300]*^T^* and [450, 0, 300]*^T^*, respectively. The yaw angles of the targets relative to ECF are −18° and 18°, respectively. Two targets are symmetric about the plane *y_e_o_e_z_e_*. The optical axis of the auxiliary camera lies in the symmetry plane *y_e_o_e_z_e_*. The error of $T_{1,2}^{\mathrm{tt}}$ obtained by Equation (22) is used to evaluate the effect of the pitch angle. Gaussian noise with σ = 0.2 pixel is added. *L*_1_ = 500 mm, *L*_2_ = 200 mm. For each level of pitch angle θ, 100 independent trials are performed.

**Figure 3:**
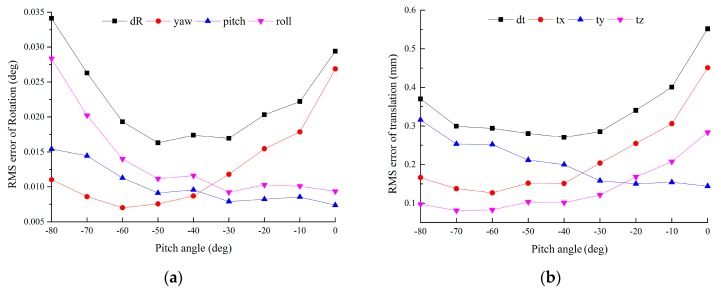
Error *vs.* the pitch angle of camera relative to the target plane: (**a**) RMS error of rotation *vs.* the pitch angle; (**b**) RMS error of translation *vs.* the pitch angle.

From Figure 3, the RMS errors of rotation and translation are roughly U-shape. When θ→−90°, the optical axis of the auxiliary camera is perpendicular to the target plane. The vanishing points approximate to infinity, which leads to higher errors. When θ→0°, the number of the extracted feature points decreases, also leads to higher errors. It is ideal to capture pair targets when θ = −40°.

### 4.2. Accuracy vs. the Yaw Angle Difference between Two Adjacent Targets 

In this experiment, ∆φ denotes the yaw angle difference between the symmetry axes of two adjacent targets. ∆φ varies according to the cameras’ distribution. We also use the error of $T_{1,2}^{\mathrm{tt}}$ calculated by Equation (22) to evaluate the effect of ∆φ.

The positions of the two targets are same with those in Section 4.1, while ∆φ varies from 0 to 85°. The two targets remain symmetric about the plane *y_e_o_e_z_e_* and the auxiliary camera lies in the symmetry plane. Gaussian noise with σ = 0.2 pixel is added. *L*_1_ = 500 mm, *L*_2_ = 200 mm. For each level, 100 independent trials are performed.

**Figure 4:**
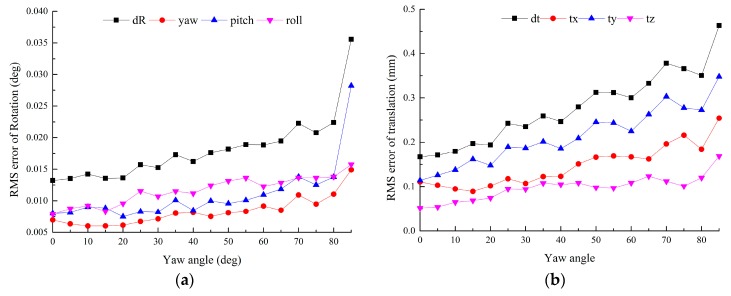
Error *vs.* the yaw angle difference between two adjacent targets: (**a**) RMS error of rotation *vs.* the yaw angle difference; (**b**) RMS error of translation *vs.* the yaw angle difference.

From Figure 4, both the error of rotation and translation rise with the increasing of ∆φ. When ∆φ > 80°, the errors increase sharply. This is because when ∆φ→90°, a group of parallel lines of each target are parallel to the image plane, thus the vanishing points approximate to infinity, which leads to great errors, so it is necessary to avoid ∆φ→90° during the calibration.

### 4.3. Accuracy vs. the Distance of Parallel Lines

In this experiment, we also use the error of $T_{1,2}^{\mathrm{tt}}$ obtained by Equation (22) to evaluate the effect of the parallel line distances. The poses of the targets are same with those in Section 4.1. The pitch angle of the auxiliary camera relative to the target plane is set to −40°. L_1_ = 500 mm, L_2_ varies from 100 mm to 400 mm. Gaussian noise with different levels is added to the image points. For each distance level, 100 independent trials are performed.

**Figure 5:**
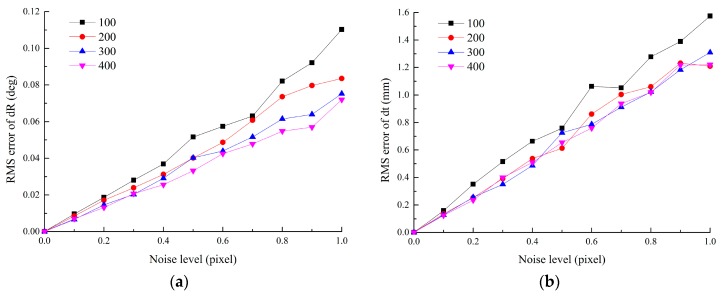
Error *vs.* the distance of parallel lines: (**a**) RMS error of rotation *vs.* the distance; (**b**) RMS error of translation *vs.* the distance.

From Figure 5, it can be seen that the error increases linearly with the noise level and decreases with the increasing distance of parallel lines. This is because the difference among slopes of intersecting lines goes up with the increasing distance of parallel lines. Calculation error of vanishing points is inversely proportional to the difference among the slopes of intersecting lines, which have been proven in [21].

## 5. Experimental Results

### 5.1. Experiment of a Use-Case

**Figure 6:**
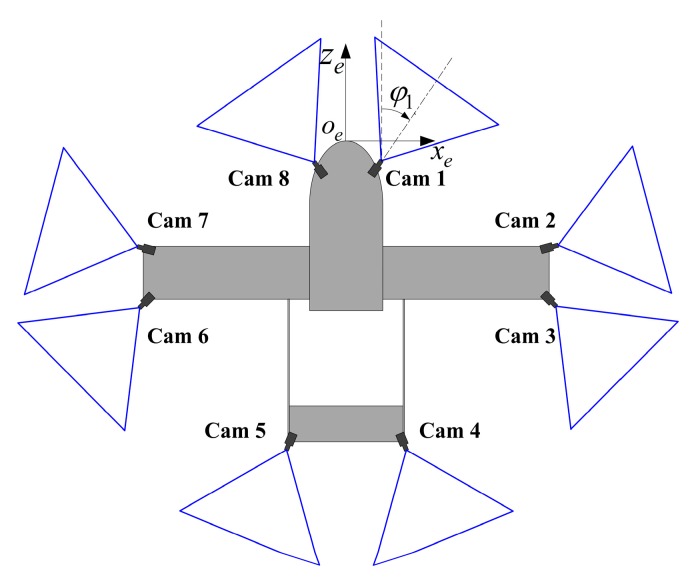
Eight cameras mounted on UAV.

Numerous situations require a system that provides a real-time view of the surroundings [28]. One of the typical cases is the operations on aerial vehicles. In this experiment, eight cameras are used to simulate a DVS mounted on an unmanned aerial vehicle (UAV), as shown in Figure 6. The proposed method is compared with other typical methods by both synthetic data and images simulated by 3ds Max software.

The intrinsic parameters of eight cameras are *f_x_* = *f_y_* = 796.44, *u*_0_ = 512, *v*_0_ = 384. The intrinsic parameters of the auxiliary camera and the image resolution are same with those in Section 4. The positions and orientations of the cameras are listed in Table 2. Each target is placed on the ground in its corresponding camera’s FOV. All the targets have the same size, *L*_1_ = 500 mm, *L*_2_ = 200 mm.

#### 5.1.1. Description of the Calibration Methods

There are many calibration methods for multiple cameras. Here five typical methods are described as follows and summarized in Table 3:

*Method 1*: This method is similar to the proposed method, except that corner points ${\tilde{p}}_{m}^{k}$ are extracted by the corner extraction engine of the J. Bouguet Camera Calibration Toolbox [23], rather than the intersections of feature lines.

*Method 2*: This method is similar to the proposed method, except that planar checkerboards with 12 × 12 grids are used as the calibration targets. The side length of each square is 50 mm.

*Method 3*: The calibration targets and the extraction of corner points ${\tilde{p}}_{m}^{k}$ are same with the proposed method. Instead of capturing neighboring target pairs, the auxiliary camera captures all the targets in one image frame, so the relative poses between targets can be computed directly.

*Method 4*: This method is similar to Method 3, except that corner points ${\tilde{p}}_{m}^{k}$ are obtained by the corner extraction method used in Method 1.

*Method 5*: This method is similar to Method 3, except that planar checkerboards of Method 2 are used as the calibration targets.

In order to illustrate the effect of the global calibration, there are two sub-methods called chainwise calibration method and global calibration method. The only difference between the two sub-methods is whether to use the global optimization in Section 3.3 or not. $T_{k1}^{\mathrm{tt}}$ (2 ≤ *k* ≤ *M*) of the chainwise method are obtained directly from multiple coordinate transformations by Equation (23), while the global calibration method is based on an additional global optimization by Equation (25).

#### 5.1.2. Synthetic Data Experiment

**Figure 7:**
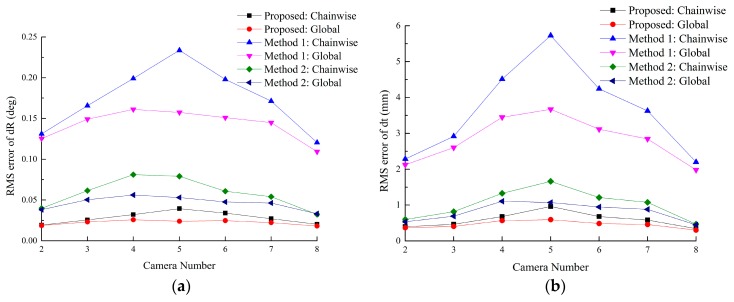

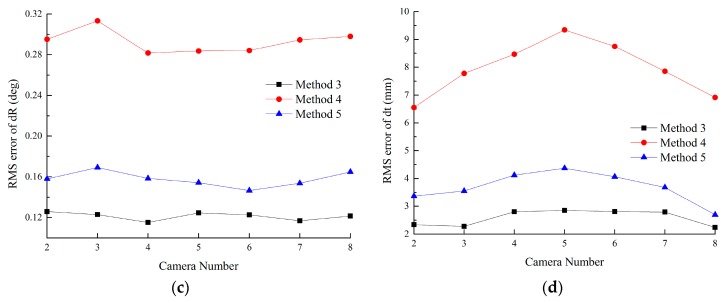
Calibration error of each method. (**a**,**b**) RMS errors of rotation vector and translation vector of the proposed method, Method 1 and 2; (**c**,**d**) RMS errors of rotation vector and translation vector of Methods 3–5.

In this experiment, the RMS error of $T_{k1}^{\mathrm{cc}}$ (2 ≤ *k* ≤ 8) is used to evaluate the accuracy. Gaussian noise with σ = 0.2 pixel is added. For each method, 100 independent trials are performed. From Figure 7, the proposed method outperforms Methods 1–5. Figure 7a,b show that the error accumulates with the coordinate transformations, and peaks at camera 5, due to the maximum times of transformations. The methods based on the constraint of ring-topologies can effectively reduce the accumulated error, especially for cameras which are far away from the reference.

Methods 3–5 do not suffer from the accumulated error issue because all the targets are visible in one image frame. However, due to limitations of image resolutions, the accuracy of the pose estimation decreases with the increasing number of the targets observed in one image.

Compared with lines-feature algorithms, points-feature algorithms are more sensitive to the image noise. Figure 7 shows that Methods 1 and 4 are worse than other methods, respectively. Further discussion is given in Section 5.3.

#### 5.1.3. Accuracy *vs.* the Image Noise Level

In this experiment, the RMS error of $T_{k1}^{\mathrm{cc}}$ (2 ≤ *k* ≤ 8) is used to evaluate the effect of the noise level. Synthetic data is same with those in Section 5.1.2. Gaussian noise from 0.0 to 1.0 pixel is added. For each noise level, 100 independent trials are performed.

**Figure 8:**
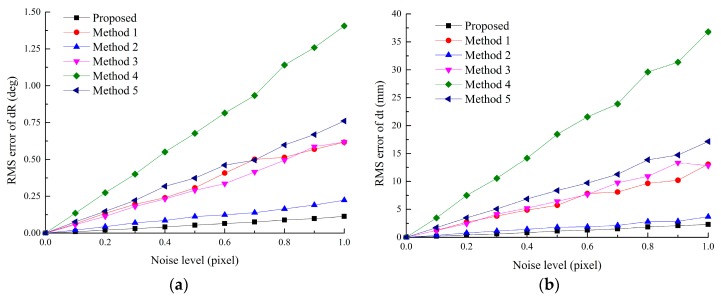
Calibration error *vs.* the noise level: (**a**) RMS error of rotation *vs.* the image noise; (**b**) RMS error of translation *vs.* the image noise.

From Figure 8, the RMS error increases linearly with the noise level. It also shows that the proposed method is superior to other methods. If the noise level of the real DVS is less than 0.5 pixels, the RMS errors of rotation and translation of all the cameras are less than 0.05 deg and 1.1 mm, respectively, which is acceptable for common applications.

#### 5.1.4. Experiments Based on Simulation Images

**Figure 9:**
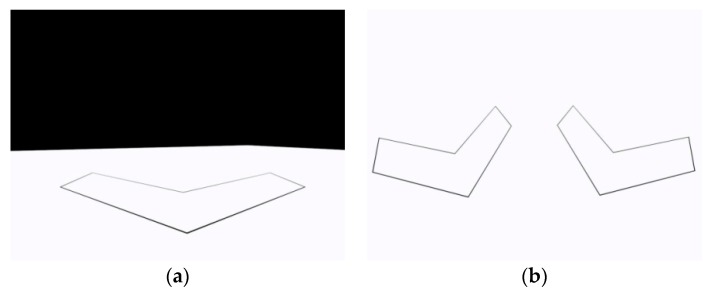
Simulation images: (**a**) Target 1 captured by camera 1; (**b**) Target 2 and Target 3 captured by the auxiliary camera.

As shown in Figure 9, we use the 3ds Max software to simulate image sequences. The parameters of the cameras and the targets are same with those in Section 5.1.2. The feature lines are obtained based on feature points extracted by Steger’s method [25]. The error of $T_{k1}^{\mathrm{cc}}$ (2 ≤ *k* ≤ 8) is used to evaluate the accuracy.

**Figure 10:**
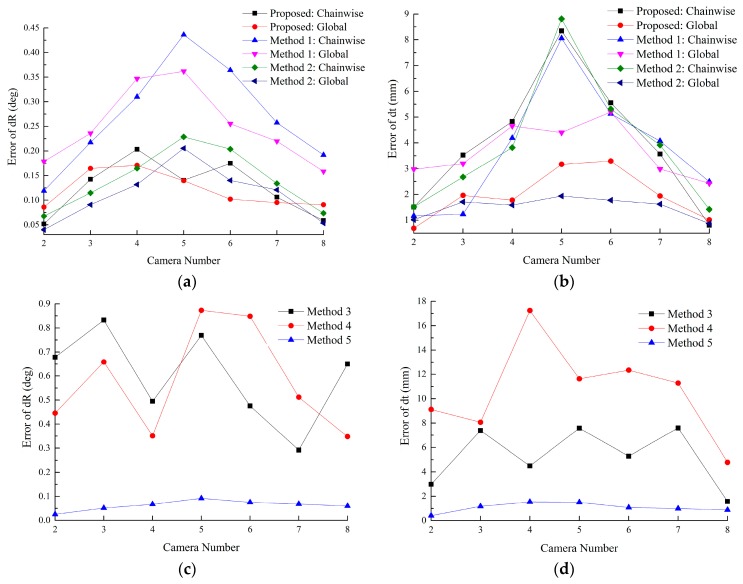
Calibration error of each method: (**a**,**b**) Errors of rotation vector and translation vector of the proposed method, Methods 1 and 2; (**c**,**d**) Errors of rotation vector and translation vector of Methods 3, 4 and 5.

Figure 10 shows that errors of rotation and translation accumulate with the increasing times of coordinate transformations. The proposed method can reduce the accumulated error due to multiple coordinate transformations. Further discussion is given in Section 5.3.

### 5.2. Real Data Experiment

**Figure 11:**
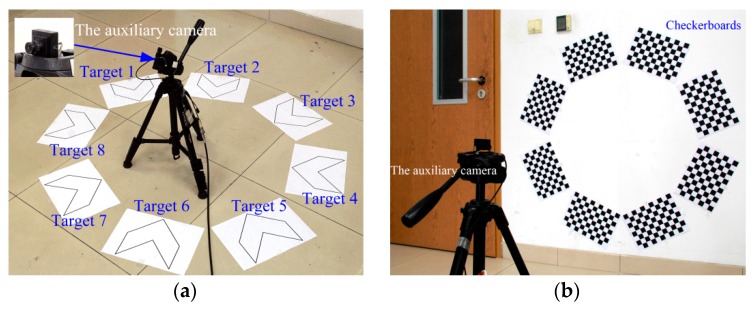
Global calibration of eight targets: (**a**) Eight targets and the auxiliary camera in the real experiment; (**b**) The auxiliary camera captures eight checkerboards in one image frame.

As shown in Figure 11a, eight targets are located in an area about 1200 mm × 1200 mm. As the relative poses of the cameras with non-overlapping FOV are mainly determined by the relative poses of the targets, the RMS errors of point pair distances between eight targets are used as calibration errors in the real experiments.

The distance of point pair $p_{m}^{k}$ and $p_{m}^{l}$ is computed according to the calibration result, and is called measurement distances, *d_m_*. The targets are also calibrated similarly by a calibrated Canon 60D digital camera. The distances of the same point pairs can be obtained in the same way and are used as the ground truth *d_t_*, due to its relatively high accuracy.

Distance error can be computed by $\Delta d=d_{m}-d_{t}$. For the proposed method, Methods 1, 3 and 4, RMS error of $\Delta d\left(P_{1}^{1}P_{1}^{k}\right),\Delta d\left(P_{2}^{1}P_{2}^{k}\right)$ and $\Delta d\left(P_{6}^{1}P_{6}^{k}\right)$ is used to evaluate the accuracy. For Methods 2 and 5, five point pairs are randomly selected and the RMS error of distance error is computed as the calibration error.

The auxiliary camera is a 1/3-in Sony CCD image sensor (ICX673) with a 3.6 mm lens. The image resolution is 720 pixel × 432 pixel. Target parameters are $L_{1}=135\text{ mm},L_{2}=70\text{ mm}.$ The image resolution of the Canon device is 1920 pixel × 1280 pixel. The intrinsic parameters of the sensors are calibrated using Bouguet’s calibration toolbox [23], as shown in Table 4.

**Figure 12:**
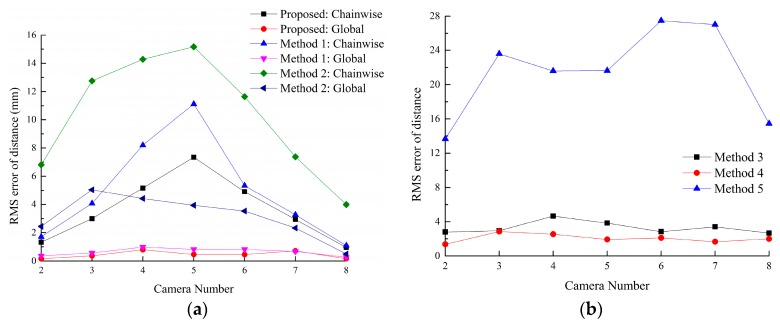
The distance error of each method. (**a**) RMS errors of the proposed method, Methods 1 and 2; (**b**) RMS errors of Methods 3, 4 and 5.

Figure 12 also shows that the proposed method achieves the best accuracy. The RMS error of point pair distances between target 5 and the reference target of the proposed method and Methods 1–5 are 0.465 mm, 0.828 mm, 3.94 mm, 3.83 mm, 1.92 mm and 21.6 mm, respectively. The proposed method is superior to other methods.

### 5.3. Discussion

Due to the restriction of image resolutions, the accuracy of the pose estimation decreases with the increasing number of the targets observed in one image. There exists a trade-off between the available features of target projections and the accumulated error from the chain of transformations. The experimental results show that the accumulated error can be effectively adjusted from the constraint of ring-topologies. For the vision sensor such as the Sony CCD sensor, capturing all the targets is not a wise choice. The benefits of direct calculation of the relative poses of the targets are cancelled out by the rise of feature extraction errors.

Moreover, it is not convenient to capture all the targets in some applications, because the auxiliary camera should be far away from the widely distributed targets. As shown in Figure 11b, in order to capture all the checkerboards in one image frame, the targets are pasted on the wall.

**Figure 13:**
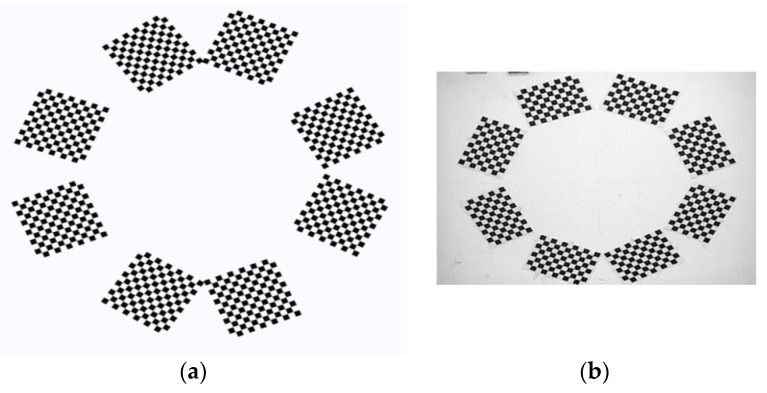
Simulation image and real image. (**a**) Checkerboards simulated by software; (**b**) Checkerboards captured by the auxiliary camera.

The results of simulation images show that the accuracy of Methods 2 and 5 is close to or even better than the proposed method. However, Methods 2 and 5 achieve the worst accuracy in the real experiments. Figure 13 shows that simulation images are very sharp and clear, which greatly benefit the corner extraction of checkerboards. However, real images could not be so ideal.

These results indicate that the proposed method is accurate and robust, especially when dealing with real images. Methods 2 and 5 are not stable against the image quality. However, there is a gap among the results of synthetic data, simulation images and real experiments. There may be some reasons for this.

Firstly, there is a measurement error during the feature extraction. In our method, the line extraction algorithm is a common-used method with acceptable accuracy and good generality. Line extraction algorithms with higher accuracy contribute to improve the accuracy, which will be further studied in the future. Secondly, the targets used in real experiments are printed on paper. They may not be strictly planar, which also leads to measurement errors.

In addition, the Sony CCD vision sensor is not a professional high-precision vision sensor, which is usually used for security cameras and radio controlled vehicles. High-resolution vision sensors can be used to improve the accuracy.

## 6. Conclusions

In this article, we have developed a new global calibration method for vision sensors in ring-topologies. Line-based calibration targets are placed in each camera’s FOV. Firstly, the relative poses of cameras and targets are initialized and refined based on the principle of vanishing features and the known line length. Next, in order to overcome small or no overlapping FOV between adjacent cameras, an auxiliary camera is used to capture neighboring targets. The relative poses of the targets is initialized in a chainwise manner, followed by nonlinear optimization to minimizing the squared distances between the observed feature lines and the re-projected corner points. Then the transformation matrix between each camera and the reference camera is determined.

The factors that affect the calibration accuracy are analyzed by synthetic data experiments. Synthetic data, simulation images and real data experiments all demonstrate that the proposed method is accurate and robust to image noise. The accumulated error can be adjusted effectively based on the constraint of ring-topologies. Real data experiments indicates that the measurement accuracy of the farthest camera by the proposed method is about 0.465 mm in an area about 1200 mm × 1200 mm. 

The poses of targets need not be known previously and can be adjusted according to the distribution of cameras. It does not need to place the targets into different positions, one placement is enough. Our method is simple and flexible and can be applied to different configurations of multiple cameras. It is well suited for the on-site calibration of widely distributed cameras. 

In this paper, we focus on the calibration of DVS in ring-topologies, which contains additional constraint. When dealing with DVS in open-topologies, accumulated errors cannot be adjusted. In addition, vanishing points approximate to infinity when feature lines are parallel to the image plane, which leads to higher errors, so the angle between parallel lines and the image plane should be in a certain range to avoid vanishing points approximate to infinity.

Restricted by hardware conditions, experiments using eight sensors mounted on an UAV are temporarily lacking. We plan to apply our method for the calibration of multiple vision sensors mounted on the vehicle in the future. Methods based on the feature lines in both indoor and outdoor environments instead of planar targets will also be investigated.

## Figures and Tables

**Table 1:** Definition of the transformation matrices.

$T_{k}^{\mathrm{tc}}$	The transformation matrix from T_k_CF to C_k_CF
$T_{ii}^{\mathrm{ta}}$	The transformation matrix from T_i_CF to A_i_CF
$T_{ji}^{\mathrm{ta}}$	The transformation matrix from T_j_CF to A_i_CF
$T_{ij}^{\mathrm{tt}}$	The transformation matrix from T_i_CF to T_j_CF
$T_{ij}^{\mathrm{cc}}$	The transformation matrix from C_i_CF to C_j_CF

**Table 2:** The positions and orientations of the cameras.

Camera ID	1	2	3	4	5	6	7	8
*x* (mm)	100	600	600	200	−200	−600	−600	−100
*y* (mm)	−150	−150	−150	−150	−150	−150	−150	−150
*z* (mm)	0	−400	−500	−1000	−1000	−500	−400	0
φ (deg)	28	64	125	158	202	235	296	332
θ (deg)	0	0	0	0	0	0	0	0
ϕ (deg)	0	0	0	0	0	0	0	0

**Table 3:** Summary of the calibration methods

Methods	Calibration Targets	Auxiliary Camera Capture
The proposed method	Line-feature targets	Neighboring target pairs
Method 1	Point-feature targets	Neighboring target pairs
Method 2	Checkerboards	Neighboring target pairs
Method 3	Line-feature targets	All targets in one image
Method 4	Point-feature targets	All targets in one image
Method 5	Checkerboards	All targets in one image

**Table 4:** Intrinsic parameters of the vision sensors

	*f_x_*	*f_y_*	*u*_0_	*v*_0_	*k*_1_	*k*_2_	*p*_1_	*P*_2_
Sony	592.73225	460.22694	343.24159	225.49825	−0.40175	0.15735	−0.00009	0.00111
Canon	1561.29373	1560.15359	972.49782	597.06160	−0.19178	0.13171	−0.00190	−0.00129

## Abbreviations

The following abbreviations are used in this manuscript:

DVS	Distributed vision Sensors
FOV	Field of view
GCF	Global coordinate frame
SIFT	Scale invariant feature transform
CCF	Camera coordinate frame
ACF	Auxiliary camera coordinate frame
ICF	Image coordinate frame
TCF	Target coordinate frame
ECF	Ground coordinate frame
RMS	root mean square
CCD	Charge coupled device

## Appendix A

A plane in a 3D space can be represented by an equation ax+by+cz+d=0. Thus, a plane may be represented by the vector $p={\left[a,b,c,d\right]}^{T}$. A 3D spatial point with homogeneous coordinates $x={\left[x_{1},x_{2},x_{3},x_{4}\right]}^{T}$ lies in the plane p if and only if $x^{T}p=0$.

Homogeneous vectors ${\left[x_{1},x_{2},x_{3},x_{4}\right]}^{T}$ such that $x_{4}\neq 0$ corresponds to finite points in ${\mathbb{R}}^{3}$. The points with last coordinate $x_{4}=0$ are known as points at infinity. The set of points at infinity can be written as $x_{\infty }={\left[x_{1},x_{2},x_{3},0\right]}^{T}$. Note that $x_{\infty }$ lies in the plane at infinity, denoted by the vector $p_{\infty }={\left[0,0,0,1\right]}^{T}$, because $x_{\infty }^{T}p_{\infty }=0$.

From Equation (5), note that $\left[-c_{i},0,a_{i},0\right]{\left[a_{i},0,c_{i},d_{i}\right]}^{T}=0,\;\left[-c_{i},0,a_{i},0\right]{\left[0,1,0,0\right]}^{T}=0$, thus the line $a_{i}x+c_{i}z+d_{i}=0,y=0$ intersects the infinite plane in the infinite point ${\left[-c_{i},0,a_{i},0\right]}^{T}$.
